# Molecular evidence of a *Trypanosoma brucei gambiense* sylvatic cycle in the human african trypanosomiasis foci of Equatorial Guinea

**DOI:** 10.3389/fmicb.2015.00765

**Published:** 2015-07-24

**Authors:** Carlos Cordon-Obras, Yasmin Fermin Rodriguez, Amalia Fernandez-Martinez, Jorge Cano, Nicolas Ndong-Mabale, Policarpo Ncogo-Ada, Pedro Ndongo-Asumu, Pilar Aparicio, Miguel Navarro, Agustin Benito, Jean-Mathieu Bart

**Affiliations:** ^1^Consejo Superior de Investigaciones Científicas, Instituto de Parasitologia y Biomedicina Lopez NeyraGranada, Spain; ^2^Centro Nacional de Medicina Tropical, Instituto de Salud Carlos IIIMadrid, Spain; ^3^Faculty of Infectious and Tropical Diseases, London School of Hygiene and Tropical MedicineLondon, UK; ^4^Centro de Referencia para el Control de Endemias, Instituto de Salud Carlos IIIMalabo, Equatorial Guinea

**Keywords:** *Trypanosoma brucei gambiense*, wild fauna, reservoir, human African trypanosomiasis, sleeping sickness, Equatorial Guinea

## Abstract

Gambiense trypanosomiasis is considered an anthroponotic disease. Consequently, control programs are generally aimed at stopping transmission of *Trypanosoma brucei gambiense* (*T. b. gambiense*) by detecting and treating human cases. However, the persistence of numerous foci despite efforts to eliminate this disease questions this strategy as unique tool to pursue the eradication. The role of animals as a reservoir of *T. b. gambiense* is still controversial, but could partly explain maintenance of the infection at hypo-endemic levels. In the present study, we evaluated the presence of *T. b. gambiense* in wild animals in Equatorial Guinea. The infection rate ranged from 0.8% in the insular focus of Luba to more than 12% in Mbini, a focus with a constant trickle of human cases. The parasite was detected in a wide range of animal species including four species never described previously as putative reservoirs. Our study comes to reinforce the hypothesis that animals may play a role in the persistence of *T. b. gambiense* transmission, being particularly relevant in low transmission settings. Under these conditions the integration of sustained vector control and medical interventions should be considered to achieve the elimination of gambiense trypanosomiasis.

## Introduction

Human African trypanosomiasis (HAT), also known as sleeping sickness, is a tropical disease caused by two subspecies of the protozoan flagellate, *Trypanosoma brucei* s.l.: *T. b. gambiense*, and *T. b. rhodesiense*. Both are closely related on a genetic level, but have important phenotypic differences regarding their epidemiology, transmission, clinical features, and distribution. The vector for *T. brucei* s.l. and other African trypanosomes is the tsetse fly (order Diptera, genus *Glossina*), but each subgenus of *Glossina* sp. has different susceptibility to diverse trypanosome species (Ashford, 2003; Kuzoe and Schofield, 2004), conditioning the distribution of both human and animal trypanosomiasis (World Health Organization, 2013).

*Trypanosoma brucei gambiense* is responsible for more than 97% of HAT cases and is spread over Central and West Africa (Simarro et al., 2012). In contrast with *T. b. rhodesiense*, which is known to circulate in domestic and wild fauna (Franco et al., 2014a), *T. b. gambiense* has traditionally been considered an anthroponosis, i.e., human beings are the main reservoir of the parasite (World Health Organization, 2013). Consistent with this assumption, control interventions based on detection and treatment of human cases have proven to be sufficient to drastically reduce the prevalence, even in the absence of vector control (Simarro et al., 2006; Franco et al., 2014b). Despite the effectiveness of these actions, virulent outbreaks have occurred over the past century after abandoning these control measures (Steverding, 2008). Thus, complete elimination was only rarely achieved in particular foci. The persistence of infection, even when no human cases were reported for years, has been attributed to different causes such as asymptomatic parasite carriers (Jamonneau et al., 2012), continuous reintroduction due to movements of both human and vector populations (Courtin et al., 2008), and inherent limitations of the surveillance system (Louis et al., 2008). An increasing number of studies have shown that domestic and wild fauna could also harbor *T. b. gambiense* even though it still remains unclear whether animals can host the parasite long enough to play an important role in the transmission of Gambiense HAT (Gibson et al., 1978; Noireau et al., 1986; Herder et al., 2002; Njiokou et al., 2006; Simo et al., 2006; Cordon-Obras et al., 2009).

Equatorial Guinea, a small country located in the Guinea Gulf, has four historical HAT foci; three in the coastal region of the continental part (Kogo, Mbini, and Campo) and one in Bioko Island (Luba) that is currently considered free of HAT (Simarro et al., 2006). Since the mid-1980s, the number of HAT cases diagnosed is very low thanks to the implementation of control campaigns based on systematic screening of endemic populations and treatment of patients. In spite of the considerable and sustained efforts, a continuous trickle of cases is reported every year in the mainland foci, Kogo, and Mbini (Franco et al., 2014a). In a previous study, we suggested that the epidemiological situation in both foci might follow different patterns. Whereas peri-domestic fauna could act as a reservoir of infection in Mbini (DNA of *T. b. gambiense* were detected in seven animals), we did not find *T. b. gambiense* in livestock from the Kogo focus (Cordon-Obras et al., 2009). In Luba, where no human cases have been reported since 1995 (Simarro et al., 2006), we were unable to detect the parasite in domestic fauna, although we found a positive tsetse fly sample, demonstrating that *T. b. gambiense* is still present in Bioko Island (Cordon-Obras et al., 2010). The persistence of infection in Kogo and Luba foci could not be explained by our data from domestic animal samples. Therefore, we hypothesized the existence of alternative epidemiological cycles. In such ecological context, where the forest represents more than 78.5% (22,000 km^2^) of the total land surface of the country, wild animals would represent potential reservoir candidates.

In the present work, employing the same molecular tools previously used (Cordon-Obras et al., 2009, 2010), we performed a molecular screening of *T. b. gambiense* in wild fauna and considered its possible role in the maintenance of the parasite in the historical foci of Equatorial Guinea. We established potential scenarios depending on the specificity of each focus, highlighting their differences, and discussed the epidemiological implications of the occurrence of a sylvatic transmission cycle for *T. b. gambiense*.

## Materials and Methods

### Study Area

**FIGURE 1:**
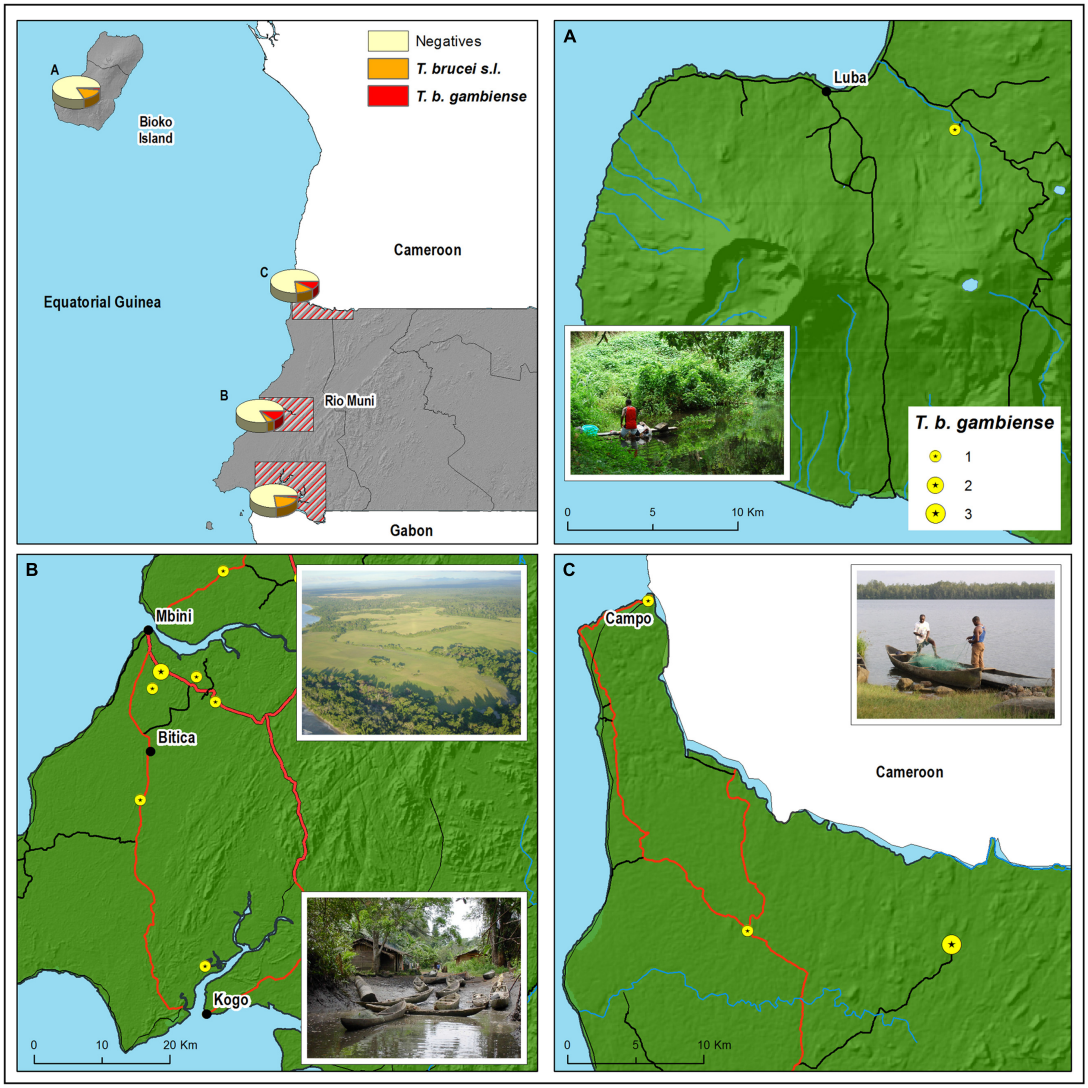
**Geographical distribution of the *Trypanosoma brucei gambiense (T. b. gambiense)*-positive animals according to the place where they were killed or trapped in **(A)** Luba, **(B)** Kogo and Mbini, and **(C)** Rio Campo.** Main and secondary roads are represented in red and black lines, respectively.

Four HAT foci from Equatorial Guinea were studied: Luba (at the southwest of Bioko Island), Rio Campo (north of the mainland region, bordering with Cameroon), Mbini (central mainland coast), and Kogo (southern mainland coast, bordering with Gabon), (**Figure 1**). Bioko Island is located more than 200 km from the mainland. Rainforest and abandoned cocoa plantations are widespread in Luba district (Simarro et al., 1990), covering a surface of 700 km^2^. Continental foci are located across the coastal region and have a typical maritime equatorial climate of four seasons. Campo extends along the Ntem River, which delineates the border between Cameroon and Equatorial Guinea, and is mostly covered by the equatorial rainforest (Simo et al., 2014). Mbini is located close to the mouth of the Wele River, whereas Kogo limits with Gabon separated by a natural boundary created by the Muni estuary. Mangrove swamp and rainforest are the predominant ecosystems in the Kogo and Mbini foci.

### Sampling Strategy

**FIGURE 2:**
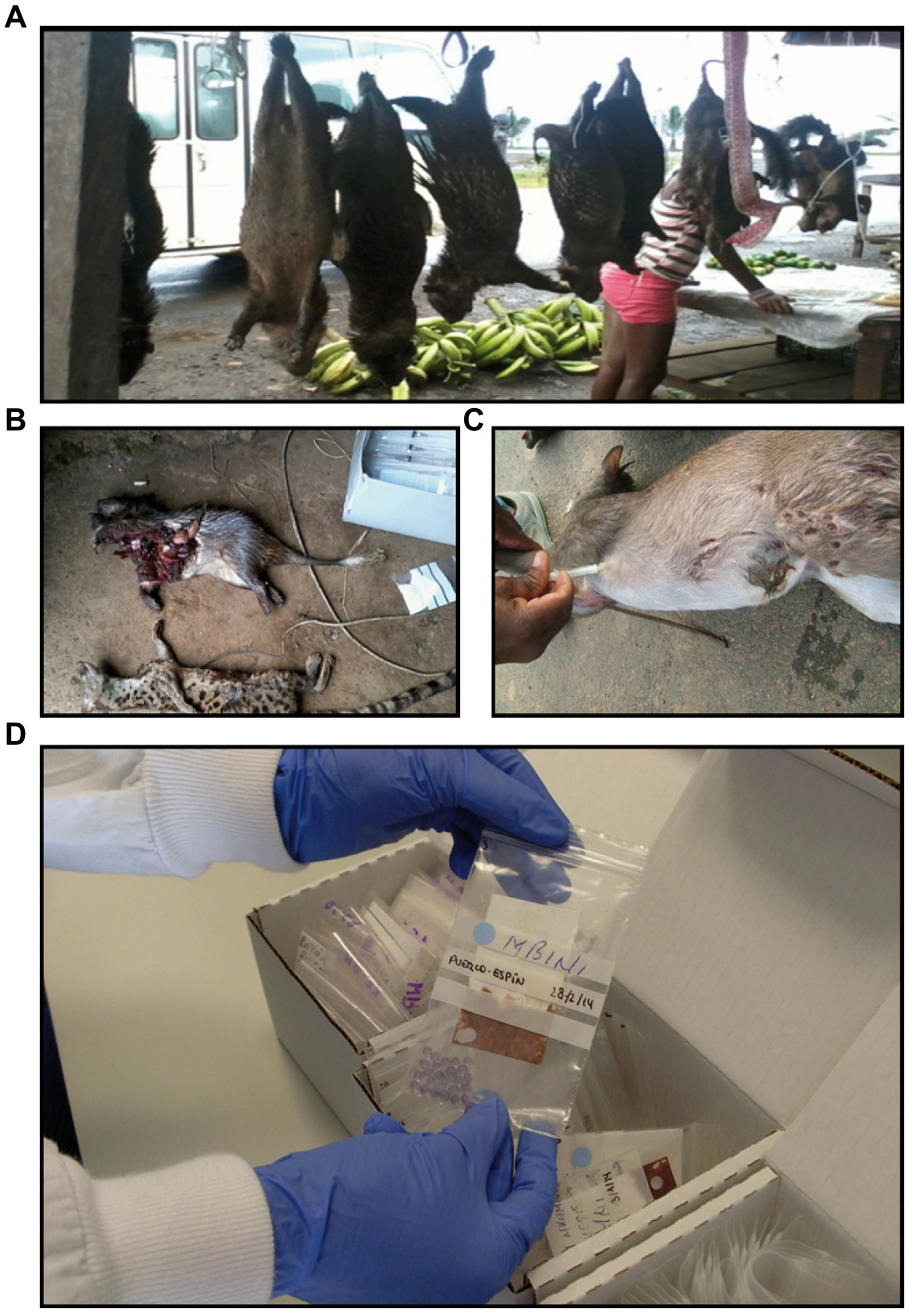
**Sampling strategy.** Blood samples from wild animals were collected from market **(A)**, or directly from the hunters **(B)**. The blood taken by venepuncture **(C)** was then spread on Whatman paper. **(D)** Filter paper with blood sample was labeled and stored separately to avoid cross-contamination.

From June 2012 to March 2014, surveys were conducted to collect blood samples from wild fauna. Samples were obtained either in local markets (**Figure 2A**) or directly from hunters and trappers (**Figure 2B**). Our team had absolutely no control over the species selected by hunters. The blood taken by venepuncture (**Figure 2C**) was spread on Whatman paper, left to dry in darkness and then stored at 4°C until processing in the reference laboratory at the National Center of Tropical Medicine, Madrid (Institute of Health Carlos III). Each filter paper was labeled to link the blood sample with the additional information recorded: hunting area, date of capture, data, and place of blood sampling (**Figure 2D**). Each sample was stored separately to avoid cross-contamination. Ethical approval was obtained by the Ministry of Health and Social Welfare and Veterinary Service from the continental region (Ministry of Agriculture, Forestry and Environment). The study adhered to the institutions’ guidelines for animal hunter game.

### Host Species Identification

Wild animals captured by hunters and trappers and brought to the village markets were identified using a systematic key (Dorst and Dandelot, 1997). Molecular confirmation was performed by cytochrome b sequencing according to the conditions previously described (Steuber et al., 2005) when the species identification was problematic. This validation was also performed on *T. b. gambiense*-positive samples in order to accurately define the possible reservoir hosts.

### Parasite Molecular Diagnosis

DNA was extracted from the samples collected employing the SpeedTools DNA Extraction kit (Biotools, Madrid, Spain) according to the manufacturer’s instructions, DNA template (1 μl) was submitted to species-specific PCR for *T. brucei* s.l. using the oligonucleotides described by Moser et al. (1989) that target the palindromic 177 bp satellite of mini-chromosomes. Positive samples for 177 bp PCR were submitted to a *T. b. gambiense* group 1-specific nested-PCR using the primers described elsewhere (Cordon-Obras et al., 2009) which amplify the known specific TgsGP gene (Berberof et al., 2001) considered to be the gold standard method for the identification of this sub-species. For both PCRs, the conditions were as follows: 1x PCR Reaction Buffer [7.5 mM Tris-HCl -pH 9-, 1.5 mM MgCl_2_, 5 mM KCl, and 2 mM (NH_4_)_2_SO_4_], 200 μM of each deoxynucleotide (dNTP), primers at 1 μM, and 1 U of Certamp Complex Enzyme Mix (Biotools, Madrid, Spain) in a final volume of 25 μl. The amplification programs were set as follows: for *T. brucei* s.l., 3 min at 95°C for initial DNA denaturation, 35 cycles of 95°C (30 s), 60°C (30 s), and 72°C (1 min), and a final extension step at 72°C (5 min). For the first reaction of *T. b. gambiense*, the fixed program was an initial denaturation step at 95°C (5 min), 35 cycles at 94°C (30 s), 55°C (1 min), and 72°C (3 min), with a final extension step at 72°C (5 min). The program for the nested reaction was identical. Negative (double distilled water) and positive [genomic DNA of *T. b. gambiense* ELIANE strain -MHOM/CI/52/ITMAP 2188- (Turner et al., 2004)] controls were systematically added to each assay. The amplification products were separated by electrophoresis in a 2% agarose gel stained with 1x RedSafe^TM^ Nucleic Acid Staining Solution (Intron Biotechnology, Inc.) and photographed under UV light. All positive samples for *T. b. gambiense* and a number of negative samples randomly selected were submitted to a second round of diagnosis performed blindly by a different researcher. All samples yielded a second positive result, confirming the robustness and reproducibility of our diagnostic method. Rigorous measures were employed to prevent contamination of the PCR reaction by establishment of a unidirectional physical workflow (pre-PCR–post-PCR).

### Statistical Analysis

Prevalence of *T. brucei* s.l. and *T. b. gambiense* infections by focus were compared through Pearson’s Chi-square test. Data analysis was conducted by using SPSS software (version 16.0.1, SPSS Inc., Chicago, IL, USA).

## Results

### Sample Collection

A total of 288 samples were collected within the four foci (121 in Luba, 66 in Mbini, 45 in Kogo, and 56 in Rio Campo) from June 2012 to March 2014. The complete animal records are provided as supplemental information (Supplementary Table S1). They included 26 animal species, 98.3% belonging to mammals, since they are the most frequent targets of hunting activities. Among mammals, the most represented orders were *Rodentia* and *Artiodactyla* (128 and 109 samples, respectively) given that the preferred preys in these foci are the African brush-tailed porcupine (*Atherurus africanus*), the African giant pouched rat (*Cricetomys gambianus*), and the hoofed blue duiker (*Cephalophus monticola*). *Primates* (29), *Carnivora* (13), and *Pholidota* (4) completed the mammal set. Four samples from reptiles and one from a bird was also analyzed.

### *Trypanosoma brucei* Infection Rate

*Trypanosoma brucei* s.l. infection in the wild fauna was ubiquitous and homogenous in the four historical HAT foci of Equatorial Guinea, with infection rates ranging from 16.5% in Luba to 24.4% in Kogo, with 19.7% in Mbini and 23.2% in Rio Campo (**Table 1**). *Rodentia* (25) and *Artiodactylia* (19) represented 77.2% of the infected animals (**Table 2**). No statistical association was found between *T. brucei* prevalence and focus (*X*^2^ = 1.84, *p*-value = 0.61).

**Table 1:** Distribution of *Trypanosoma brucei* s.l. and *Trypanosoma brucei gambiense* (*T. b. gambiense*) among wild fauna in different foci of Equatorial Guinea.

Focus	*T. brucei* s.l. no. (%)	*T. b. gambiense* no. (%)
Luba	20/121 (16.5)	1/121 (0.8)
Mbini	13/66 (19.7)	8/66 (12.1)
Kogo	11/45 (24.4)	1/45 (2.2)
Rio Campo	13/56 (23.2)	5/56 (8.9)
**Total**	**57/288 (19.8)**	**15/288 (5.2)**

**Table 2:** Distribution of *T. brucei* s.l. and *T. b. gambiense* according to animal species.

Focus	Species		Intra-species Prevalence (%)	
	Common name	Scientific name	*T. brucei s.l.*	*T. b. gambiense*
**Luba**	***Carnivora***			
	Small-spotted genet	*Genetta servalina*	1/2 (50)	-
	***Rodentia***			
	Red-legged sun squirrel	*Heliosciurus rufobrachium^∗^*	3/16 (18.8)	1/16 (6.3)
	Brush-tailed porcupine	*Atherurus africanus*	5/34 (14.7)	-
	African giant pouched rat	*Cricetomys gambianus*	3/25 (12)	-
	***Artiodactyla***			
	Blue Duiker	*Cephalophus monticola*	7/35 (20)	-
	***Reptiles***			
	African Rock Python	*Python sebae*	1/1 (100)	-
**Mbini**	***Primates***			
	Red-tailed monkey	*Cercopithecus ascanius^∗^*	1/2 (50)	1/2 (50)
	Grey-cheeked mangabey	*Lophocebus albigena^∗^*	1/1 (100)	1/1 (100)
	Greater spot-nosed monkey	*Cercopithecus nictitans*	1/5 (20)	-
	***Carnivora***			
	Small-spotted genet	*Genetta servalina*	1/1 (100)	1/1 (100)
	African palm civet	*Nandinia binotata*	2/3 (66.7)	1/3 (33.3)
	***Rodentia***			
	Brush-tailed porcupine	*Atherurus africanus*	3/14 (21.4)	3/14 (21.4)
	African giant pouched rat	*Cricetomys gambianus*	1/6 (16.7)	1/6 (16.7)
	***Artiodactyla***			
	Blue Duiker	*Cephalophus monticola*	3/19 (15.8)	-
**Kogo**	***Carnivora***			
	Small-spotted genet	*Genetta servalina*	1/1 (100)	-
	***Rodentia***			
	Brush-tailed porcupine	*Atherurus africanus*	4/5 (80)	-
	***Artiodactyla***			
	Blue Duiker	*Cephalophus monticola*	5/22 (22.7)	1/22 (4.5)
	Red River Hog	*Potamochoerus porcus*	1/1 (100)	-
**Rio Campo**	***Primates***			
	Greater spot-nosed monkey	*Cercopithecus nictitans*	3/4 (75)	1 /4 (25)
	***Carnivora***			
	African Lisang	*Poiana richardsonii^∗^*	1/*2* (50)	1/*2* (50)
	***Rodentia***			
	Brush-tailed porcupine	*Atherurus africanus*	4/11 (36.4)	1/11 (9.1)
	African giant pouched rat	*Cricetomys gambianus*	2/7 (28.6)	-
	***Artiodactyla***			
	Blue Duiker	*Cephalophus monticola*	3/9 (33.3)	2/9 (22.2)

*^∗^Species detected as putative *T. b. gambiense* reservoir for the first time in this study.*

A total of 15 samples were positive for the TgsGP nested-PCR. To ensure the specificity of these bands, the PCR products were sequenced. They all presented 100% homology with the TgsGP gene (DAL 972 reference strain). *T. b. gambiense* was detected in all foci, but with a heterogeneous pattern. Infection rate of *T. b. gambiense* was 12.1 and 8.8%, in Mbini, and Rio Campo, respectively, and significantly lower in Luba (0.8%) and Kogo (2.2%; *X*^2^ = 13.48, *p*-value <0.01). No statistical association was found between prevalence of *T. brucei* or *T. b. gambiense* and the host order (*X*^2^ = 7.91, *p*-value = 0.34 and *X*^2^ = 11.85, *p*-value = 0.1, for *T. brucei* and *T. b. gambiense*, respectively).

Overall, 10 mammals species were found to harbor *T. b. gambiense*, belonging to four orders (*Rodentia, Artiodactyla, Carnivora*, and *Primates*; **Table 2**). Similarly to *T. brucei* s.l. infection, 60% of the *T. b. gambiense* cases were distributed among rodents and artiodactyls; carnivores and primates represented the remaining 40%. Among those positives for *T. b. gambiense*, we identified four species so far never reported as putative reservoirs of this parasite*;* these were *Heliosciurus rufobrachium* (Red-legged sun squirrel), *Cercopithecus ascanius* (red-tailed monkey), *Lophocebus albigena* (gray-cheeked mangabey), and *Poiana richardsonii* (African lisang).

## Discussion

The four historical foci of Equatorial Guinea depict the diversity of epidemiological settings. In the past, Luba was the most important focus of HAT in this country, reporting hundreds of cases per year (Simarro et al., 1990). The implementation of control activities based on active detection and systematic treatment of patients in 1985 drove to the elimination of the human disease one decade later (Simarro et al., 2006). Kogo and Mbini always had a marginal importance compared to Luba in terms of HAT incidence. However, despite similar control measures were implemented the complete elimination has not been yet achieved in the mainland foci. On the other hand, Campo has historically reported few cases (10 cases since 2000 according to Equatorial Guinea Ministry of Health) even in the absence of control interventions in the Guinean part of the focus (Simo et al., 2014). One might speculate that the isolation of Bioko Island would have contributed to the success of control interventions, whereas factors such as population movements, reintroduction of infected vectors, and insufficient coverage in the campaigns of active screening might be leading to a steady occurrence of cases in the mainland foci.

In this study, we demonstrate the presence of *T. b. gambiense* in different species of wild fauna in all the four endemic HAT foci of Equatorial Guinea. These findings support our previous works, where we concluded that diverse eco-epidemiological scenarios, involving different animal reservoirs, might underlie the variety of transmission patterns for *T. b. gambiense* in Equatorial Guinea.

We already reported that *T. b. gambiense* was present in domestic livestock from Mbini but not Kogo (Cordon-Obras et al., 2009). According to these results, we speculated on the occurrence of a peri-domestic transmission cycle in Mbini, involving livestock often circulating close to populations. In the present study, while the *T. brucei* s.l. infection rate is similar in all foci, we observed a significantly higher prevalence of *T. b. gambiense* in wild fauna of Mbini compared to Kogo. These data suggest an additional transmission activity in a sylvatic cycle along with the peri-domestic cycle previously hypothesized (Cordon-Obras et al., 2009). It is noteworthy that the prevalence of *T. b. gambiense* infection in wild fauna in Mbini focus was higher than previously reported in peri-domestic livestock (12.1% vs. 2%; Cordon-Obras et al., 2009), which could be explained by a greater preference of vector population for wild fauna or simply a greater availability of wildlife versus livestock. Further studies aiming to describe the feeding preferences of tsetse flies will be conducted to clear up this question. In Kogo, where new HAT cases are actually rare, *T. b. gambiense* has been detected in only one wild animal out of 45 screened, indicating that (i) a sylvatic cycle can occur in this focus, but (ii) parasite transmission level seems low.

Campo focus, shared by Equatorial Guinea and Cameroon, has particular epidemiological features. Although this focus has remained active in both sides of the border, only a handful of HAT cases have been reported over the last years in the Guinean part. Subsequently, unlike the other foci, active screening has not been undertaken in a regular basis in Campo. Some studies have reported the presence of *T. b. gambiense* in animals and the occurrence of human infections in the Cameroonian side of the border, even though at low level (Morlais et al., 1998; Penchenier et al., 1999; Herder et al., 2002). Our data reveal 8.9% of infection rate in wild fauna, above the prevalence reported in wild (0.6%; Njiokou et al., 2006) and domestic (4.4%; Njiokou et al., 2010) animals on the Cameroonian side. The different molecular markers used in the screening or the diverse epidemiological patterns can in part explain these contrasted results. It is noteworthy that in the Cameroonian focus of Fontem, it was described a high *T. b. gambiense* prevalence in pigs (14.8%; Simo et al., 2006), suggesting different epidemiological scenarios between foci from Cameroon as we noticed in Equatorial Guinea. According to our findings we might conclude that *T. b. gambiense* is mainly circulating in a sylvatic cycle in Campo, and the absence of human cases is likely due to the low population density in the area. However, a plan to communicate both sides of Ntem river by road (Economic Community of Central African States source, see http://www.ceeac-eccas.org) and the development of Campo municipality in the Guinean part would undoubtedly result in an increase of human presence in the area and in turn, a greater risk of exposure to *T. b. gambiense* infection.

The Luba focus has not reported autochthonous HAT cases since 1995 (Simarro et al., 2006). However, in a recent publication we demonstrated the persistence of *T. b. gambiense* in tsetse flies (Cordon-Obras et al., 2010). Given that no vertical transmission of the parasite has been reported in the tsetse fly and the apparent lack of infection in the local livestock, we suggested that a wild cycle could be the responsible for the maintenance of *T. b. gambiense*, even after the absence of HAT cases for decades (Simarro et al., 2006). In the present work, we found a positive sample of *T. b. gambiense* amongst the blood samples gathered from wild animals (0.8% prevalence). The epidemiological scenario of Luba seems to be quite similar to Kogo focus, where HAT cases are now rarely reported, *T. b. gambiense* is apparently absent in domestic fauna and this parasite is found at low prevalence in wild fauna. All these data together strongly suggest the existence of a sylvatic cycle with low activity in both foci which may hinder a definitive eradication of the disease.

Our data also revealed homogenous prevalence of *T. brucei* s.l. in all the studied foci, but this pattern is not observed in *T. b. gambiense*. This could be explained by the fact that control measures taken over human reservoirs and the HAT epidemiological features of each focus affect *T. b. gambiense*, but not *T. b. brucei* which is, besides, much more easily acquired and transmitted by tsetse flies (Kuzoe and Schofield, 2004).

As sampling is biased due to the methods, the species that host the parasite are similar to those found in other African foci (Njiokou et al., 2006), i.e., those that are targets of hunting and trapping. In spite of this bias, we identified some species that have never been previously reported as reservoirs of *T. b. gambiense*. In addition, we cannot rule out that more animal species may harbor *T. b. gambiense*. Indeed, there is no apparent association between the rate of infection and taxonomic order in either *T. b. brucei* or *T. b. gambiense*, suggesting that these parasites can adapt to a wide range of species with no evident preference. This is consistent with previous findings that point out the ability of trypanosomes to proliferate in several species in both *in vivo* and *in vitro* experiments (Bitter et al., 1998; Herder et al., 2002; Njiokou et al., 2006; Cordon-Obras et al., 2013). Besides, it is well known that most of the other human pathogenic kinetoplastids like *T. b. rhodesiense, T. cruzi*, or *Leishmania* sp. are able to circulate in a wide range of animal species (Sobrino et al., 2008; Oda et al., 2014; Zanet et al., 2014).

Despite the provided evidences, we assume that detecting *T. b. gambiense* in wild fauna does not definitively prove that these animals represent relevant reservoirs for the maintenance of the parasites that are then able to infect humans. Nevertheless, epidemiological arguments such as the higher significant prevalence in Mbini (the most active HAT focus) and Campo (where no control measures were implemented), the previously detected presence of the parasite in tsetse flies in Luba (but not in humans or domestic fauna; Cordon-Obras et al., 2010), and the fact that most of the animals had already been detected previously as being positive for *T. b. gambiense* (Njiokou et al., 2006), support the existence of sylvatic cycles. If that is correct, it would remain to be elucidated whether both animal and human cycles are linked. To answer this question, we will further analyze by microsatellite approach the samples used in this study and others from HAT patients concurrently isolated from the same foci. The accurate genetic identification of the same populations circulating in both cycles would provide a strong argument in support of our hypothesis.

The lack of an appropriate description of possible reservoirs of *T. b. gambiense* is a fundamental drawback in the design of control strategies. Wild fauna is a link in the epidemiological chain that is much more difficult to control, and interventions directed against vector would probably be the way to interrupt parasite transmission. Animal reservoirs often mask the infection of a parasitic or viral disease between outbreak periods. Over the 20th century, HAT progression has followed a similar pattern (Steverding, 2008), with alternating periods of low incidence and virulent outbreaks. The intensive control efforts focused on human patients have been always very effective to control the disease, but rarely sufficient to definitively eliminate it in any focus. Recently, based on mathematical models of gambiense HAT transmission involving humans, peri-domestic and wild animals in the Cameroonian focus of Bipindi, a study suggested the crucial role of animals in the maintenance of *T. b. gambiense* (Funk et al., 2013). In Equatorial Guinea, if alternative reservoirs are ruled out and neglected in control programs, *T. b. gambiense* could be maintained in the wild cycle as has probably occurred in Luba, and may lead to virulent outbreaks in the future as has happened in the past (Steverding, 2008). As previously addressed by other authors (Gouteux and Artzrouni, 1996; Solano et al., 2013), vector control will be essential to a complete elimination and we strongly recommend its introduction in eradication programs to complement the active screening focused in the human patients.

## Conflict of Interest Statement

The authors declare that the research was conducted in the absence of any commercial or financial relationships that could be construed as a potential conflict of interest.
